# Physiological Characterization and Comparative Transcriptome Analysis of White and Green Leaves of *Ananas comosus* var. *bracteatus*

**DOI:** 10.1371/journal.pone.0169838

**Published:** 2017-01-17

**Authors:** Xia Li, Surapathrudu Kanakala, Yehua He, Xiaolan Zhong, Sanmiao Yu, Ruixue Li, Lingxia Sun, Jun Ma

**Affiliations:** 1 College of Landscape Architecture of Sichuan Agricultural University, Chengdu, Sichuan, China; 2 Institute of Plant Protection, Agricultural Research Organization, The Volcani Center, Beit Dagan, Israel; 3 Horticultural Biotechnology College of South China Agricultural University, Guangzhou, Guangdong, China; NARO Institute of Fruit Tree Science, JAPAN

## Abstract

Leaf coloration is one of the most important and attractive characteristics of *Ananas comosus* var. *bracteatus*. The chimeric character is not stable during the *in vitro* tissue culturing. Many regenerated plants lost economic values for the loss of the chimeric character of leaves. In order to reveal the molecular mechanisms involved in the albino phenotype of the leaf cells, the physiological and transcriptional differences between complete white (CWh) and green (CGr) leaf cells of *A*. *comosus* var. *bracteatus* were analyzed. A total of 1,431 differentially expressed unigenes (DEGs) in CGr and CWh leaves were identified using RNA-seq. A comparison to the COG, GO and KEGG annotations revealed DEGs involved in chlorophyll biosynthesis, chloroplast development and photosynthesis. Furthermore, the measurement of main precursors of chlorophyll in the CWh leaves confirmed that the rate-limiting step in chlorophyll biosynthesis, and thus the cause of the albino phenotype of the white cells, was the conversion of pyrrole porphobilinogen (PBG) to uroporphyrinogen III (Uro III). The enzyme activity of porphobilinogen deaminase (PBGD) and uroporporphyrinogn III synthase (UROS), which catalyze the transition of PBG to Uro III, was significantly decreased in the CWh leaves. Our data showed the transcriptional differences between the CWh and CGr plants and characterized key steps in chlorophyll biosynthesis of the CWh leaves. These results contribute to our understanding of the mechanisms and regulation of pigment biosynthesis in the CWh leaf cells of *A*. *comosus* var. *bracteatus*.

## Introduction

Pineapple is an herbaceous perennial monocot that belongs to the *Bromeliaceae* family. Plants of this family are native to South America and are cultivated commercially for their fruit, and the high-quality silk fiber of their stem and leaves [1, 2]. These plants are a source of bromelain, which is a proteolytic enzyme complex used in the meat industry for its health benefits [3]. To date, a large number of secondary metabolites have been synthesized from *Ananas* leaves and fruit infusions [4–8]. Moreover, pineapple carries out crassulacean acid metabolism (CAM), also known as CAM photosynthesis, and more recently the pineapple genome sequence and the expression and regulations of the genes associated with CAM were analyzed [9].

*A*. *comosus* var. *bracteatus* is an important ornamental plant due to its colorful leaves and decorative red fruits. The colorful leaves consist of normal green cells and albino white cells. *A*. *comosus* var. *bracteatus* is self-incompatible, and thus tissue culture is a fast and effective method of cultivation. However, the chimeric character is not stable during tissue culturing. Only about 1% of the regenerated plants were chimera plants. More than 80% of the regenerated plants were CGr and CWh plants, which are of low economic value because they lack chimera leaves [10]. It is of significant importance to understand the mechanism of chimera formation in order to enhance the stability of the chimera character. Leaf color mutants are the best material for investigation of the chlorophyll (Chl) metabolic pathway, chloroplast development, gene regulation, and the photosynthesis system [11, 12]. Changes in concentration of Chl in leaves will change the color of leaves [13]. To date, the transcriptional variation between the two types of cells and the molecular mechanisms of the albino cells have not been understood. We have observed by microscope that the chimera leaves were composed of two types of cells, the normal green cells and the albino white cells. However, the normal green cells and the albino white cells were intermixed both in the green and white parts of the chimera leaves. The CGr and CWh plants derived via tissue culture are more typical presentation of the normal green and albino white cells respectively. The leaf color of the regenerated plants of CWh or CGr plants was the same as that of the mother plant [10]. The CWh and CGr plants are stable and typical in leaf color. In this study, we used the CGr and CWh plants as material to study the physiological and transcriptional differences between the two types of leaf cells.

Previous studies focused on the genetic diversity of the genus *Ananas*. Data on expressed sequence tags (ESTs), sequencing of *A*. *comosus* var. *comosus* roots, fruit and aerial tissues [14], green mature fruits [15], and nematode infected gall have been performed [16] have been published. Transcriptome sequencing of the leaf, stem and root of *A*. *comosus* var. *bracteatus* was conducted by Ma et al. [17]. Recently, genome sequencing of *A*. *comosus* (L.) Merr. has been published and the evolution of the CAM photosynthesis was shown [9]. However, the mechanism behind the albino appearance of the leaf cells and the development of the chimera plant in *A*. *comosus* var. *comosus* was not well understood.

In the present study, we undertook *de novo* transcriptome sequencing of CGr and CWh leaves of *A*. *comosus* var. *bracteatus*. To our knowledge, this is the first comparative transcriptome characterization of *A*. *comosus* var. *bracteatus*. A total of 1,431 DEGs were identified by RNA-seq. Among them, 858 were the up-regulated genes and 573 were the down-regulated genes. Functional annotation of these DEGs provided an overview of the transcriptional variation between the CWh and CGr leaves. The top three DEG-enriched pathways were photosynthesis, porphyrin and chlorophyll metabolism, and carotenoid biosynthesis. The conversion of PBG to Uro III was the rate-limiting step of chlorophyll biosynthesis in CWh leaves, and *hemC* was the key gene in this reaction as identified by transcriptome sequencing, quantitative analysis of concentration of the main precursors of Chl biosynthesis, and analysis of activity of the 5-aminolevulinic acid dehydratase (ALAD), PBGD and UROS. The expression of genes involved in chlorophyll biosynthesis was validated by quantitative reverse transcription polymerase chain reaction (qRT-PCR). These results provide a valuable resource for further genetic and genomic studies on leaf color formation in *A*. *comosus* var. *bracteatus* and other plant species.

## Materials and Methods

### Plant materials

The CWh and CGr *Ananas comosus* var. *bracteatus* tissue culture plants were derived from chimera plants using our previously published protocol [11]. At the stage of ten to twelve leaves, the palnts were used as source of samples for transcriptome sequencing and physiological detections. The chimera plants were obtained from a garden in Zhanjiang, Guangdong Province (coordinates 21°12′N 110°24′E), China. No specific permissions were required for these locations, because the study did not include field study. The studies did not involve endangered or protected species.

### Measurement of chlorophyll and carotenoid contents

The CGr and CWh leaves were selected for chlorophyll and carotenoid measurements. The pigment (Chl *a*, Chl *b*, and carotenoid) concentrations were measured using the Holm equation and the method as previously described [18].

### Assessment of chlorophyll biosynthetic precursors

The concentration of 5-aminolevulinic acid (ALA) was detected according to the method of Dei et al. [19]. The concentration of PBG was measured suing the method previously described by Bogorad et al. [20]. The concentrations of Uro III and coprogen III (Cop III) were measured according to the method of Czarnecki et al. [21].

### Enzyme activity determination

The activity of ALAD was analyzed following the method previously described by Mauzerall and Granick [22]. The activity of PBGD was detected according to the protocol of Riminton [23]. For measurement of enzymatic activity of UROS, the leaves were homogenized in 2 ml buffer (pH 8.2, 0.05 M Tris-HCl, 8 mM MaCl_2_, and 5 mM mercaptoethanol). The homogenate was centrifuged at 10^3^ × g for 10 min. A total of 0.9 ml of the supernatant was mixed with 0.1 ml 4 M PBG. After incubation at 37°C for 5 min, 30 μl of 5% I_2_ (v/v) was added to the solution. The solution was incubated at 37°C for 5 min, and 50 μl 1% Na_2_S_2_O_3_ and 0.1 ml 1% trichloroacetic acid were added. Then the solution was centrifuged at 8,000 g for 10 min. The absorption value of the supernatant was measured at 553 nm.

### RNA extraction, cDNA library creation and Illumina sequencing

Total RNA was extracted from CWh and CGr plants using an RNeasy plus Micro Kit (Qiagen, Hilden, Germany) following the recommendations of the manufacturer. RNA integrity was assessed using the RNA Nano 6000 Assay Kit and the Agilent Bio analyzer 2100 system (Agilent Technologies, CA, USA). Each RNA sample was subjected to DN*ase* digestion (Takara) to remove any remaining DNA. Enrichment of mRNA, fragment interruption, addition of adapters, size selection, PCR amplification and RNA-Seq were performed using the Illumina HiSeq 2500 sequencing platform, Beijing Biomarker Technologies, Beijing, China. The remaining RNA was used for real-time quantitative PCR (qPCR) verification.

### Sequence assembly and differential expression analysis

The raw reads were cleaned by removing adapter sequences and low quality sequences. The high quality reads were annotated by BLAST best hit mapping to *Ananas comosus* var. *bracteatus* representative unigenes (NCBI BioProject: PRJNA317274, NCBI BioSample: SAMN04604740) [17]. Quantitative expression was determined by mapping of all Illumina reads against *A*. *comosus* var. *bracteatus* representative coding sequences using Bowtie and cutting the best hit for each read. Zero counts were treated as true 0. Expression was normalized to Reads Per Kilobase per Million mappable reads (RPKM). The false discovery rate (FDR) <0.01 and fold change ≥2 were chosen as the threshold of expression to discriminate background transcription.

DESeq [24] was used with an upper quartile normalization method [25] to test for differential expression between CWh and CGr cells. The Benjamini and Hochberg method was applied to the list of resulting P-values to control FDR [26]. Genes that were differentially expressed between CWh and CGr cells (FDR<0.01) were then separated into CWh or CGr expression using the log2 fold change of CWh/CGr. The functional annotation of identified genes was based on the Mapman pathways (http://mapman.gabipd.org/) with manual corrections, further refinement [27, 28] and Phytozome (http://www.phytozome.net/).

### Functional annotation of DEGs

The DEGs were compared against the NCBI nr, nt and Swiss-Prot databases. The Swiss-Prot BLAST results were imported into BLAST2GO to retrieve gene functions to be determined and they were compared with GO terms [29]. The DEG sequences were also aligned with the COG database to predict and classify functions. The Kyoto Encyclopedia of Genes and Genomes (KEGG) pathways were used to assign DEGs with the online KEGG automatic annotation server (KAAS), http://www.genome.jp/kegg/kaas/. The functions of enzymes were annotated by UniProt (http://www.uniprot.org/).

### Real-time quantitative reverse transcription (qRT)-PCR

For qPCR analysis, total RNA from CWh and CGr plants from *in vitro* cultures were extracted as described above. RNA (2 μg) was reverse transcribed using a primeScript® RT reagent Kit with gDNA Eraser (Takara) according to the manufacturer’s instructions. The gene-specific primers were designed based on the DEGs. Primer sequences are listed in S1 Table. qPCR DNA amplification and analysis were carried out using the SYBR® Premix Ex TaqTM II (Tli RNase H Plus) kit (Takara) with an ABI Prism®7900HT real-time PCR System (ABI). qPCR conditions were 5 min at 95°C, followed by 40 cycles of 5 s at 95°C, 5 s at 60°C, and 10 s at 72°C, followed by 65°C to 95°C for melting curve detection. Data were analyzed using the Pfaffl method [30]. Four internal controls, namely, ubiquitin, histone H1, elongation factor 1 alpha and α-tubulin, were used as the internal control. Experiments were performed for three biological repeats.

## Results

### Deriving of CGr and CWh plants via tissue culture

**Fig 1:**
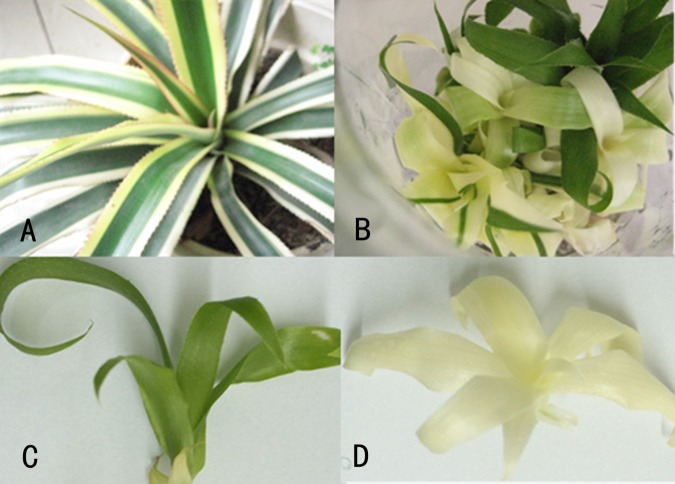
Derivation of complete green (CGr) and complete white (CWh) plants via tissue culture. (A) the 2-year old plant with chimera leaves. (B) the shoots generated from the callus were derived from stem of a chimera plant that shows the color variation of the regenerated shoots. (C) CGr plant derived via *in vitro* tissue culture. (D) CWh plant derived via *in vitro* tissue culture.

The stem of the chimera plant (Fig 1A) was used for tissue cultuing for fast generation of shoots. After 30 d of culturing, a callus was induced from the stem explant. After 30 d of sub-culturing, adventitious shoots generated from the callus. Every piece of callus could generate many shoots. However, the shoots that regenerated included CGr shoots, CWh shoots and chimera shoots (Fig 1B). The CGr and CWh plants accounted for about 40% of the regenerated plants respectively. Only about 1% of the regenerated shoots were chimera plants same as the mother plants. The CGr (Fig 1C) and CWh (Fig 1D) shoots were used as samples for this study.

### Chlorophyll and carotenoid content analysis of the CWh and CGr leaves

**Fig 2:**
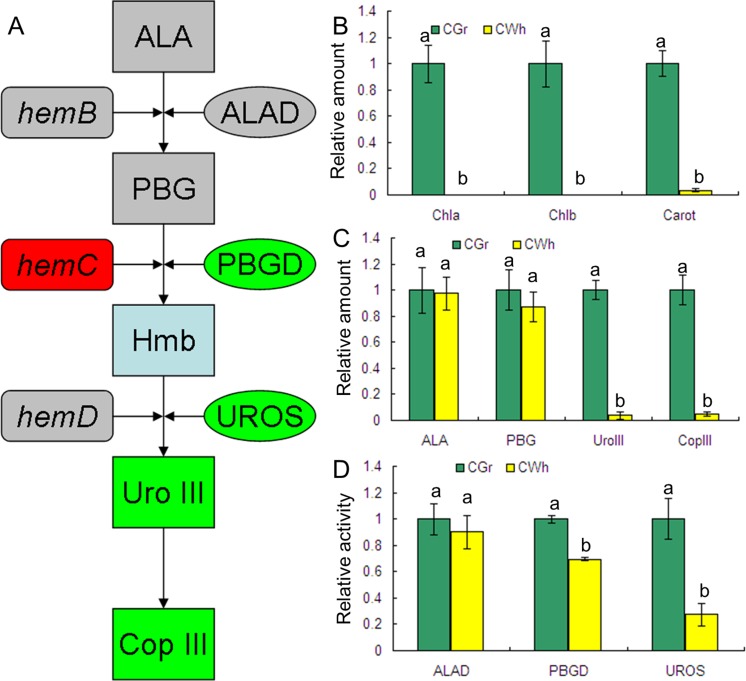
Identification of the rate-limiting step of chlorophyll biosynthesis in CWh leaves of *A*. *comosus* var. *bracteatus*. (A) Schematic view of the metabolism pathway of ALA to Cop III. The rectangle shows precursors of chlorophyll. The rounded rectangle shows the gene encoding protein catalyzing the reaction of the precursors. The oval shows the enzyme catalyzing the reaction of the precursors. The gray color means there was no significant difference between the CWh and CGr leaves. The red color means significantly up-regulated in the CWh leaves. The green color means significantly down-regulated in the CWh leaves. (B) The variation of relative amount of chlorophyll and carotenoids between CWh and CGr leaves. (C) The relative concentration of main chlorophyll precursors. (D) Activity differences of ALAD, PBGD and UROS between CWh and CGr leaf cells. B-D shows the relative values of CWh leaves which use the value of CGr leaves as control and calculated as 1. Different letters in columns indicate statistically significant differences (*P*<0.01) according to a T-test.

The concentrations of Chl a, Chl b and Chl (a + b) of CWh leaves were below the detection limits of the method used in this study (Fig 2B). This result means that the concentration of chlorophyll in CWh leaves is very low, and that the CWh leaves are composed of albino white cells containing very little chlorophyll. In contrast of the CWh leaves, Chl a, Chl b and Chl (a + b) were present in CGr leaves at relatively high concentration. These measurements confirmed that the CWh and CGr leaves were the typical presentations of the albino white cells and the normal green cells, respectively, in the *A*. *comosus* var. *bracteatus* leaves. The concentration of carotenoid of the CGr leaves was found to be about thirty times that in the CWh leaves.

### Assessment of chlorophyll biosynthetic precursors

Since the Chl contents in CWh leaves were significantly lower than in CGr leaves, the concentration of main precursors of Chl biosynthesis were measured to determine the rate-limiting step of Chl biosynthesis. The concentrations of each precursor in the two types of leaves are shown in Fig 2C. There were no significant differences in ALA and PBG concentrations in CWh and CGr leaves. However, the concentration of Uro III and Cop III in the CGr leaves was about thirty times the concentration found in the CWh leaves. This finding suggests that the conversion of PBG to Uro III is likely the rate-limiting step of Chl biosynthesis in the albino white leaf cells of *A*. *comosus* var. *bracteatus*.

### Comparison of enzyme activities in CWh and CGr plants

ALAD catalyzes conversion of ALA to PBG. PBGD and UROS function in the catalysis of PBG to Uro III. Hydroxymethylbilane (Hmb) is the unstable intermediate product of PBG catalyzed by PBGD (Fig 2A). Because concentration of Chl in the CWh leaves is significantly lower than in the CGr leaves (Fig 2B), and the first significant difference in the concentration of chlorophyll precursors was detected at Uro III (Fig 2C), we determined the activity of ALAD, PGBD and UROS. The results showed that there was no significant difference in the enzyme activity of ALAD in the CGr and CWh leaves while the activities of PBGD and UROS in the CWh leaves were significantly lower than in the CGr leaves (Fig 2D). These data suggest that conversion of PBG to Uro III is a rate-limiting step of chlorophyll biosynthesis in the CWh leaves, and that the decrease in enzyme activities of PBGD and UROS resulted in suppressed formation of Uro III.

### Transcriptome sequencing and comparison with the reference genome

The comparative analysis of the sequenced transcriptome and the reference genome [9, 17, 31] could reveal the common and unique features in the *Ananas* species. The CGr and CWh shoots, derived from the callus of the chimera plant, represent the normal green and albino white cells of the chimera leaves, respectively. The transcriptomes of both CGr and CWh leaves were determined by RNA-Seq using two complementary technologies to gain quantitative gene expression information. The RNA-Seq libraries of green and white plants were sequenced with Illumina HiSeq 2500 technology.

The raw reads were filtered and clean reads were mapped to the reference sequence database derived from *Ananas* [9, 17, 31]. After the removal of adaptor sequences and the exclusion of contaminated or short reads, 11 million reads each with the Cycle Q30 percentages 88.44% and 88.42% were yielded. Additionally, 79.29% or 78.94% of reads matched the reference sequence database and approximately 92% of the mapped reads were unique mapped reads (Table 1). These results demonstrated the effectiveness of Illumina sequencing technology in rapidly capturing a large portion of the transcriptome. In the reference sequence database, 858 were detected as differentially up-regulated in CWh plants and 573 were detected as differentially down-regulated in CWh plants (Table 1). Among these DEGs, 336 genes were dramatically different (161 >+2 and 175 <-2 log2-fold change). Details of the DEGs are listed in the S2 Table. To facilitate the access to and use of the transcriptome sequencing data, the data have been deposited in NCBI with BioProject number: PRJNA317275.

**Table 1:** Sequencing, mapping, and assembly statistics for complete green and white plants.

Read mapping	Complete White plant(CWh)	Complete green plant(CGr)
No. of total reads	11,815,346	11,778,566
Cycle Q20 percentage	100.00	100.00
Q30 percentage	88.42	88.44
Mappable reads (%)	9,326,483(78.94%)	9,339,392(79.29%)
Unique mapped reads	8,621,259(92.44%)	8,624,388(92.34%)
Multiple mapped reads	705,224(7.56%)	715,004(7.66%)
No. of contigs in assembly	41,052	
Differentially up-regulated	858	
Differentially down-regulated	573	

### The functional annotation of the DEGs

The DEGs were blasted to the non-redundant protein (nr) database, NCBI non-redundant nucleotide sequence (nt) database, UniProt/Swiss-Prot, Gene Ontology (GO), UniProt/TrEMBL, Kyoto Encyclopedia of Genes and Genomes (KEGG) database, Cluster of Orthologous Groups of proteins (COG) by BLAST software. The annotations of the DEGs are presented in Table 2. According to the BLASTx results, 1,283 (89.7%) unigenes had homologues proteins in the nr protein database, and 1,149 (80.3%) showed significant matches in the Nt database, and 1,064 (74.4%) unigenes had similarity to proteins in the Swiss-Port database. There were 1,060 (74.1%) DEGs found with GO annotation, 282 (19.7%) DEGs with KEGG annotation and 553 (38.6%) DEGs with COG annotation. Of the 1,431 unigenes, approximately 90% of the DEGs matched to known genes, which confirmed the high quality of the transcriptome sequence.

**Table 2:** Functional annotation of the DEGs between CGr and CWh plant.

Type	nr	nt	SwissPort	GO	TrEMBL	KEGG	COG
**No. of DEGs**	1,283	1,149	1,064	1,060	1,300	282	553

**Fig 3:**
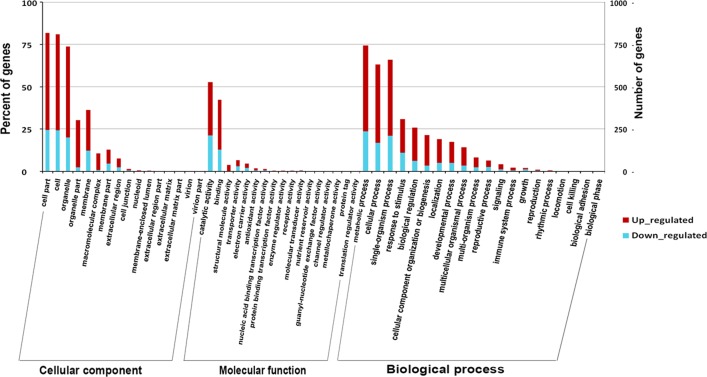
Functional annotation of DEGs based on gene ontology (GO) categorization. GO analysis was performed at the level of three main categories (cellular component, molecular function and biological process).

The functions of the DEGs were classified with the GO database (Fig 3, S3 Table). A total of 1,060 DEGs were divided into three ontologies: cellular component, molecular function and biological process. Of these, the majority of the GO terms were assigned to cellular components (43.8%), followed by biological processes (41.1%) and molecular functions (15.1%).

**Fig 4:**
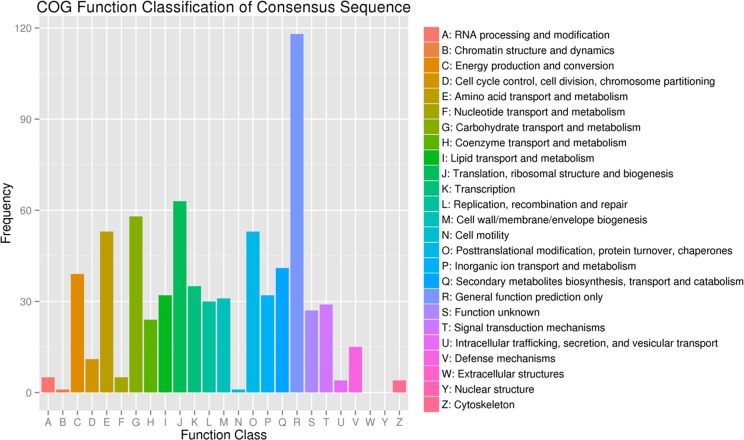
Clusters of orthologous group (COG) classification. In total, 535 DEGs with nr hits were grouped into 25 COG classifications.

The percentages of DEGs in nucleoids, protein binding transcription factor activity, locomotion, and cell killing were obviously higher than the percentages of unigenes in these categories. This result indicated that the DEGs were highly enriched in these aspects, and that more attention should be paid to these aspects in future studies. In all categories, there were more up-regulated genes than down-regulated genes. More than twice as many genes were up-regulated than were down-regulated across most categories. It is suggested that the transcription levels in the CWh leaves were enhanced, compared to the CGr plants, due to the compensatory mechanism caused by the lack of chlorophyll and thus suppressed photosynthesis. Based on the nr annotation, all the DEGs were subjected to a search against the COG database for functional annotation and classification. In total, 553 DEGs could be assigned to the COG classifications and were divided into 25 specific categories as shown(Fig 4, S4 Table).

### GO and KEGG pathway enrichment analysis of DEGs

To uncover the biological roles of the DEGs between *A*. *comosus* var. *bracteatus* CGr and CWh plants, we performed a top GO analysis. The numbers of up- and down-regulated DEGs were further compared between the CWh and CGr plants using three ontologies: cellular component, molecular function and biological process, and the details of this analysis are listed in the S5 Table.

The most enriched terms (KS <10E-10) of the cellular component category were involved in the chloroplast thylakoid membrane (107 DEGs), chloroplast stroma (154 genes), chloroplast envelope (130 DEGs), ribosome (43 DEGs), and chloroplast thylakoid lumen (29 DEGs). These results suggested that the DEGs in the structure and function of chloroplasts were enriched.

**Fig 5:**
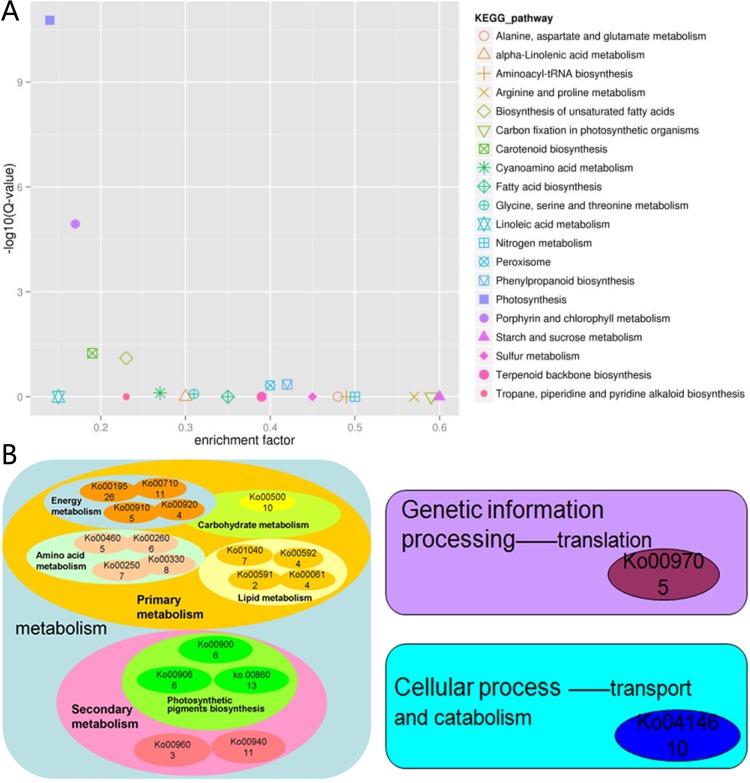
The first twenty DEGs enriched in the KEGG pathway. (A) The enrichment factor and –log10 (Q-value) of the first twenty enriched pathways. The smaller of the enrichment factors and the bigger of –log10 (Q-value) indicated the pathway with more DEG enrichment. (B) Function cluster of the first twenty enriched pathways. The number in the small elliptical frame shows the number of DEGs annotated in the pathway.

The KEGG database can categorize gene functions with an emphasis on biochemical pathways. A BLASTx search against the KEGG protein database was made on the DEGs. A total of 208 DEGs were grouped into 82 pathways (S6 Table). The most significantly enriched pathway was photosynthesis (Ko. 00195, 26 DEGs, 12.5%), followed by porphyrin and chlorophyll metabolism (Ko. 00860, 13 DEGs, 6.25%), and carotenoid biosynthesis (Ko.00906, 6 DEGs, 2.88%) pathway (Fig 5A). An overview of the function cluster of the first twenty enriched KEGG pathways is shown in Fig 5B. This cluster of results indicated that the transcriptional differences between CWh and CGr plants were found mainly in the metabolism process of the plants, especially in the primary metabolism. The lack of energy and primary metabolites resulted in suppression of the secondary metabolism.

### DEGs annotated to chlorophyll biosynthesis between CGr and CWh plants

The DEGs annotated in photosynthetic pigment biosynthesis-related pathways were imported into MapMan software (http://mapman.gabipd.org) to obtain a transcriptional overview of the pathways related to leaf color (S1 Fig). Most of the DEGs annotated in the chlorophyll biosynthesis pathway and carotenoid biosynthesis were up-regulated in the CWh plants. However, the DEGs annotated in the upstream pathways were down-regulated in the CWh plants (S1 Fig, S7 Table).

**Fig 6:**
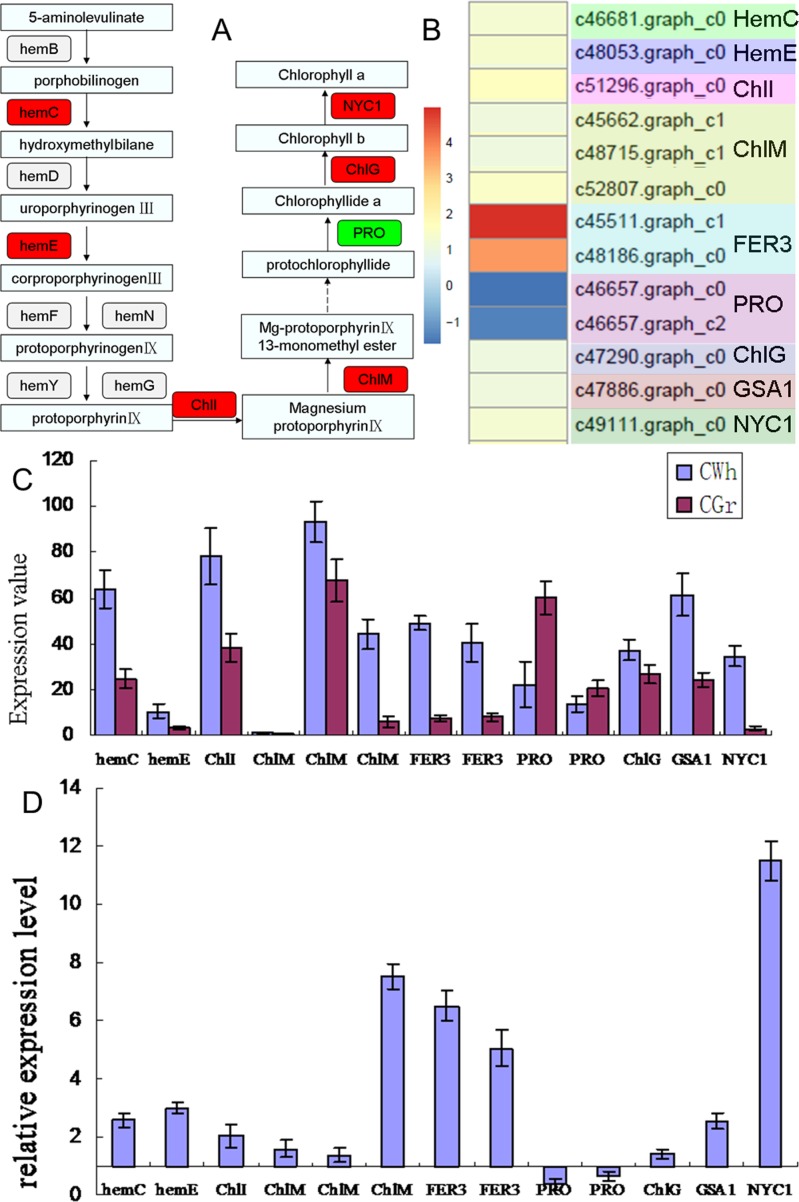
Expression level of the candidate DEGs related to chlorophyll biosynthesis. (A) Schema of the main pathway of chlorophyll biosynthesis marked with the DEGs detected by RNA-seq. Red color marked the gene detected up-regulated in the CWh leaves; green color marked the gene detected down-regulated in the CWh leaves. (B) Differential expression value (log2CWh/CGr) of 13 DEGs annotated to porphyrin and chlorophyll metabolism pathway identified by RNA-seq. (C) Expression values of the 13 DEGs in the CGr and CWh leaves detected by qRT-PCR. (D) The relative expression level of the 13 DEGs detected by qRT-PCR. The scale shows the ratio calculated with the expression level of the CGr plant as a reference and expression level of the CWh plant as a test. The ratio value means the gene expression level in the CWh plant was N times that of the CGr plant.

Fig 6A summarizes the main reactions in chlorophyll biosynthesis and the DEGs identified in this study that encode proteins function in the catalysis of these reactions. Six genes were found to be up-regulated in the CWh leaves while only one gene was down-regulated. The differential expression values of the DEGs are shown in Fig 6B and S8 Table. These genes play roles in the transition of glutamate to ma-protoporphyrin IX 13-monomethylester. Glutamate-1-semialdehyde 2,1-aminomutase (EC5.4.3.8, c47886.graph_c0, GSA1, hemL) is up-regulated in the CWh leaves. It is a transaminase converting glutamate 1-semialdehyde (GSA) to 5-aminolevulinate (ALA). The ALA is subsequently converted to PBG, Uro III, Cop III, protoporphyrin IX, protochlorophyllide and then to chlorophyll b and chlorophyll a in a serious of enzymatic steps. Among these enzymes, hydroxymethylbilane synthase (EC2.5.1.61, c46681.graph_c0, hemC), which catalyzes the tetrapolymerization of the monopyrrole PBG into the hydroxymethylbilane pre-uroporphyrinogen in several discrete steps, was found to be up-regulated in the CWh leaves. This is in contrast to the finding of decreased enzyme activity of its encoding protein (PBGD). This suggests that suppression of chlorophyll biosynthesis in the CWh leaves was caused by decreased function of PBGD protein encoded by the *hemC* gene. The lack of enzyme activity resulted in a compensatory increase of hemC gene expression in the CWh leaves. As a consequence, the uroporphyrinogen decarboxylase 1 (EC4.1.1.37, c48053.graph_c0, hemE) was also up-regulated in the CWh leaves. The latter catalyzes decarboxylation of four acetate groups of uroporphyrinogen- III to yield coproporphyrinogen- III. The next up-regulated gene was the magnesium-protoporphyrin IX chelatase subunit (magnesium chelatase, ChlI) that encodes the protein (EC6.6.1.1) catalyzing chelation of magnesium and protoporphyrin IX to form magnesium-protoporphyrin IX. Subsequently, magnesium-protoporphyrin IX is converted to magnesium-protoporphyrin IX 13-monomethylester, catalyzed by magnesium protoporphyrin IX methyltransferase (EC2.1.1.11, ChlM). The encoding gene of ChlM (c46681.graph_c0) was found to be up-regulated in the CWh leaves. However, the gene encoding protochlorophyllide reductase (EC1.3.1.33, c46657.graph_c0; c46657.graph_c2, PRO), which catalyzes the light-dependent trans-reduction of the D-ring of protochlorophyllide was down-regulated in the CWh leaves. This was the only gene related to the biosynthesis of chlorophyll that was found to be down-regulated in the CWh leaves (Fig 6B). This gene could be another key gene involved in chlorophyll biosynthesis in the CWh leaves. The chlorophyll synthase (EC2.5.1.62, c47290.graph_c0, ChlG), catalyzes chlorophyllide a to chlorophyll a conversion, while chlorophyll (ide) b reductase (EC1.1.1.294, c49111.graph_c0, NYC1) enzymecatalyzes the first step in the conversion of chlorophyll b to chlorophyll a; the genes encoding these enzymes were also up-regulated in the CWh leaves.

The maximally up-regulated gene was ferritin (EC1.16.3.1, c45511.graph_c1 and c48186.graph_c0, FER3). Ferritin is involved in oxidation of Fe (++) to Fe (+++) and promotes incorporation of the latter into proteins such as apotransferrin and lactoferrin (Fig 6B). It plays a role in porphyrin biosynthesis pathway which competes with chlorophyll biosynthesis. The up-regulated expression of *FER3* may suppress the chlorophyll biosynthesis in the CWh leaves.

The expression levels of the thirteen DEGs detected by RNA-seq in chlorophyll biosynthesis were detected by qPCR (Fig 6C). The relative expression patterns of the thirteen genes are shown in Fig 6D. Among the 13 detected genes, except two PRO genes, the remaining DEGs were up-regulated in CWh plants. qRT-PCR results concur with the RNA-seq data, confirming the reliability of our sequencing data.

## Discussion

It has been reported that changes in concentration of Chl will change the color of the leaves [13]. Studies have shown that tissue culture is a good method for generation of *A*. *comosus* var. *bracteatus* with a high regeneration rate. However, the leaf color of the regenerated shoots varies greatly from the stock plant [11]. Typically, the CGr plant loses its ornamental value and the CWh plant dies after two or three months of culture. The variation in leaf color essentially prevents the application of tissue cultures in *A*. *comosus* var. *bracteatus* production. Therefore, it is important to reveal the differences between the green and white leaves and the mechanisms that determine the albino phenotype of leaf cells.

The biosynthesis and accumulation of pigments in plant leaves are regulated by various intrinsic and extrinsic factors [32–35]. In our study, the concentrations of Chl in CWh and CGr plants were significantly distinct. In practice it is important to enhance the photosynthetic properties of tissue culture plantlets because chlorophyll biosynthesis is often suppressed [33–38]. This study found that concentrations of Chl a and Chl b in the CWh leaves were too low to be detected. This finding suggested that the CWh leaves show a typical presentation of albino leaf cells and might be an ideal sample for studing the mechanism of albinism.

The rate-limiting steps in Chl biosynthesis are different in various plant species. In this study, we found no significant differences in the concentrations of ALA and PBG. However, the concentrations of Uro III and Cop III in the CWh leaves were significantly lower than in the CGr leaves. This suggested that the conversion of PBG to Uro III was the rate-limiting step in Chl biosynthesis in the CWh leaves of *A*. *comosus*. var. *bracteatus*.

Previous studies have confirmed that the loss of green color in leaves is caused by suppressed expression of genes involved in the biosynthesis of Chl or development of chloroplasts [39–42]. Based on our findings of the rate-limiting step in this study, we can deduce that *hemC* and *hemD*, which encoding proteins catalyze PBG to Uro III, potentially playing important roles in the biosynthesis of Chl. Significant reduction of enzyme activity of *hemC* and *hemD* encoding proteins PBGD and UROS in the CWh leaves further confirms that the decrease of chlorophyll biosynthesis in the albino leaves is likely caused by inhibition of enzyme activity of hemC protein. However, the expression of *hemC* was up-regulated in the CWh leaves compared to the CGr leaves as demonstrated in both transcriptome sequencing and qPCR analyses. This indicated that although the expression level of *hemC* gene in CWh leaves was enhanced, the function of hemC protein was actually suppressed. It is suggested that the function of hemC encoding protein is likely the key factor in Chl biosynthesis in the albino leaf cells. There could be other factors (single nucleotide polymorphisms, methylation, long non-coding RNA, and microRNAs) that can detrimentally affect the function of hemC protein. The translation and modification of hemC protein and regulations of hemC protein activity need to be analyzed in future studies.

The other important gene related to Chl biosynthesis in CWh leaves is *PRO* gene. It is the only down-regulated gene involved in Chl biosynthesis detected by RNA-seq. The decreased expression of *PRO* gene is likely to further suppress the biosynthesis of Chl in CWh leaves. The function and transcriptional regulation of *PRO* gene need to be analyzed in future study to reveal the albino phenotype of the white leaf cells.

Using the key genes (*hemC*, *PRO*) screened in this study as molecular markers, we can further study the arrangement rules of the normal green cells and albino white cells in the chimera leaves during the development of the chimera plants. This study can help the understanding of the formation mechanism of the chimera plants and can provide theoretical basis for the regulation of the stability of the chimera character.

In recent years, transcriptional profiling provided valuable information about novel genes at various developmental stages or in different physiological conditions of a cell [43, 44]. Moreover, RNA-seq identified unique genes involved in various biological pathways in different colored plant tissues [45, 46]. Since the CWh and CGr plants were typical representatives with the normal green and albino white cells of the chimera leaves, we decided to use the CWh and CGr leaves to study the transcriptional differences between these two types of cells.

The transcriptome analysis revealed that the maximally DEG enriched KEGG pathways included photosynthesis, porphyrin and chlorophyll metabolism, and carotenoid biosynthesis. The blast results of GO database showed that the DEGs were enriched in the structure and function of the chloroplasts, which may be caused by the lack of chlorophyll. This indicated that the transcriptional differences between the CGr and CWh leaves play roles in biosynthesis of photosynthetic pigments and the development of chloroplasts, which then cause the differences in photosynthesis. Decreased photosynthesis results in the suppression of primary metabolism and subsequently the secondary metabolism. This mechanism may explain the death of CWh plants after only two to three months of culturing.

The qPCR results confirmed that the transcriptome sequence analysis in this study was reliable. These results can provide a basis for an overview of the transcription variation between the CGr and CWh plants, and it can further assist future studies of the molecular mechanisms underlying the formation of chimera plants.

This study provided physiological and comparative transcriptome analysis of CWh and CGr plants. These results will serve as an excellent platform for future studies seeking to understand the physiological and transcriptional differences between CWh and CGr leaves and the molecular mechanisms behind the albino phenotype of leaf cells in *A*. *comosus* var. *bracteatus*.

## Supporting Information

S1 Fig MapMan illustration of enriched pathways related to photosynthetic pigment biosynthesis. Sequenced expression data were analyzed by the MapMan software. Among the photosynthetic biosynthesis pathways, porphyrin and chlorophyll metabolism and carotenoid pathway showed striking enrichment. Red color in square boxes represents up-regulation and blue represents repressed expression in complete white plant compare to complete green plant.
(TIF)

S1 Table List of primers used in this experiment. (DOC)

S2 Table Expression details and functional annotations of DEGs between the CWh and CGr plant. (XLS)

S3 Table Statistic of the functional annotation results of DEGs based on gene ontology (GO) categorization. (XLS)

S4 Table Cluster and statistic of orthologous groups (COG) classification. (XLS)

S5 Table The topGO categories enrichment results of the DEGs between the Wh and Gr plant. (XLS)

S6 Table The enrichment results of DEGs based on KEGG database. (XLS)

S7 Table The expression details and functional annotations of DEGs analyzed by MapMan software. (XLS)

S8 Table Differential expression (log2) of DEGs identified by transcriptome sequence annotated in the porphyrin and chlorophyll metabolism pathway. (DOCX)
