# Supplementary figures and images for: Physiological Characterization and Comparative Transcriptome Analysis of White and Green Leaves of Ananas comosus var. bracteatus
Source: PLoS One. 2017 Jan 17;12(1):e0169838. doi: 10.1371/journal.pone.0169838 (PMC5240938; doi:10.1371/journal.pone.0169838)

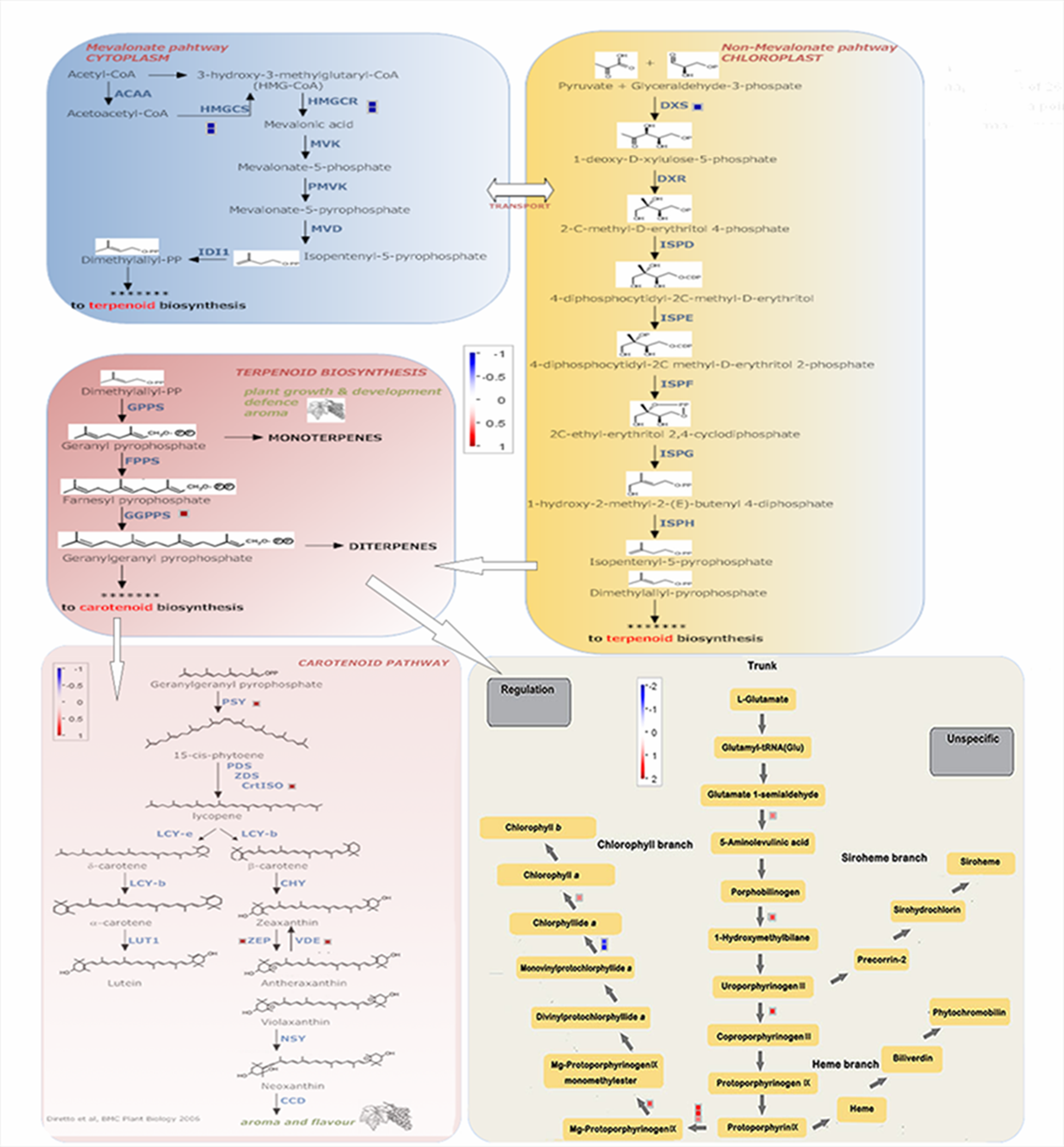

Supplement: S1 Fig — Sequenced expression data were analyzed by the MapMan software. Among the photosynthetic biosynthesis pathways, porphyrin and chlorophyll metabolism and carotenoid pathway showed striking enrichment. Red color in square boxes represents up-regulation and blue represents repressed expression in complete white plant compare to complete green plant. (TIF) [file pone.0169838.s001.tif]
