# Supplementary material for: Physiological Characterization and Comparative Transcriptome Analysis of White and Green Leaves of Ananas comosus var. bracteatus
Source: PLoS One. 2017 Jan 17;12(1):e0169838. doi: 10.1371/journal.pone.0169838 (PMC5240938; doi:10.1371/journal.pone.0169838)
Supplement: S1 Table — (DOC) [file pone.0169838.s002.doc]

Table S1 sequences of primers used in this experiment

| Gene ID | Gene name | Forward primer (5’-3’) | Reverse primer (5’-3’) |
| --- | --- | --- | --- |
| c44200.graph_c1  c40810.graph_c0  c48812.graph_c2  c44491.graph_c0  c46681.graph_c0  c48053.graph_c0  c45662.graph_c1  c51296.graph_c0  c48715.graph_c1  c45511.graph_c1  c48186.graph_c0  c46657.graph_c0  c46657.graph_c2  c47290.graph_c0  c47886.graph_c0  c49111.graph_c0  c52807.graph_c0 | elongation factor1 alpha  Histone H1  ubiquitin  α-tubulin  HemC  HemE  ChlD  ChlI  ChlM  FER3  FER3  PRO  PRO  ChlG  GSA1  NYC1  ChlH | TAAGAATGTTGCTGTGAAG  TATAGCGAAGCATATTGAA  ATAGCAGCCAAGTTCAAT  CCATACAATAGCGTCCTA  AGATGTCTGAATATGTTG  GGACAATGAGCAAGAGAA  TTCCCGCCTTTCCAGAAGT  TACGAGATGAGTGTTGGT  CAAGGTGCTATCGGAATA  AAGCATTGAAGGAGATAG  TGGTTGCTATGATTACTT  GACTGACGAAATTCATAGG  GGATAATGTTGCTCTGAGA  TCTACGCAGTTCTACTTC  TCTCTACGCAGTTCTACT  AGAACCAGATAGCATCAG  AGAACCTTGAGAGGCTTA | CCTGAGAGGTGAAGTTAG  TTTGGCAGTAAAGTTCTT  TTCATTCCATTCAGCATCT  ATAGCCTCGTTATCCAATA  CCTTCCTAATGTCTTATG  CATCTACTATACCACCTTCAG  CACCTGTGCGAGCCTCTT  GGATAATCAGACTTGTTCAGA  ATCAAGTGTCTCAAGGAAT  CTACGGATAACTGAGTAAT  GAACATTCCTCTTATATGC  TCTGAACAAGAACTCCAT  CGTTTGTTCTGGTATTTCATA  TTACGAGGCAGTTACTTG  TTACGAGGCAGTTACTTG  TCTCAATCCATCCACATT  CGGACTGATTGGTAACTAC |
