# Supplementary material for: Physiological Characterization and Comparative Transcriptome Analysis of White and Green Leaves of Ananas comosus var. bracteatus
Source: PLoS One. 2017 Jan 17;12(1):e0169838. doi: 10.1371/journal.pone.0169838 (PMC5240938; doi:10.1371/journal.pone.0169838)
Supplement: S8 Table — (DOCX) [file pone.0169838.s009.docx]

| Gene | Symble | Accession # | Log_2_ (Wh/Gr) |
| --- | --- | --- | --- |
| Porphobilinogen deaminase | HemC | c46681.graph_c0 | 1.30 |
| Uroporphyrinogen decarboxylase 1 | HemE | c48053.graph_c0 | 1.40 |
| Magnesium-chelatase subunit | ChlD | c45662.graph_c1 | 1.11 |
| Magnesium-chelatase subunit | ChlI | c51296.graph_c0 | 1.67 |
| Magnesium protoporphyrin IX methyltransferase | ChlM | c48715.graph_c1 | 1.04 |
| Ferritin-3 | FER3 | c45511.graph_c1  c48186.graph_c0 | 4.97  3.71 |
| Protochlorophyllide reductase | PRO | c46657.graph_c0;  c46657.graph_c2 | -1.59  -1.34 |
| Chlorophyll synthase | ChlG | c47290.graph_c0 | 1.09 |
| Glutamate-1-semialdehyde 2,1-aminomutase | GSA1 | c47886.graph_c0 | 1.12 |
| Chlorophyll(ide) b reductase | NYC1 | c49111.graph_c0 | 1.31 |
| Magnesium-chelatase subunit | ChlH | c52807.graph_c0 | 1.51 |

**S8 Table.** Differential expression (log2) of DEGs identified by transcriptome sequence annotated in the porphyrin and chlorophyll metabolism pathway.
